# Untargeted Metabolomic Analysis Reveals Plasma Differences between Mares with Endometritis and Healthy Ones

**DOI:** 10.3390/ani14131933

**Published:** 2024-06-29

**Authors:** Xijun Zhang, Yujin Gao, Zhanhai Mai, Yina Li, Jiamian Wang, Xingxu Zhao, Yong Zhang

**Affiliations:** 1 College of Veterinary Medicine, Gansu Agricultural University, Lanzhou 730070, China; zhangxijun0325@163.com (X.Z.); gyj1234561202@163.com (Y.G.); mzh881231@126.com (Z.M.); lyn9097@163.com (Y.L.);; 2 Gansu Key Laboratory of Animal Generational Physiology and Reproductive Regulation, Lanzhou 730070, China

**Keywords:** endometritis, mare, plasma, metabolomics, biomarkers

## Abstract

**Simple Summary:**

Endometritis is a common reproductive system disease in mares that severely threatens their fertility, causing significant economic losses. Currently, the clinical diagnosis of endometritis in mares relies on case-by-case clinical examinations, which is particularly inefficient in large farms. This study revealed differences in plasma metabolites between mares with endometritis and healthy mares, identifying a total of 25 differentially enriched metabolites. Among these, 10 specific metabolites were highlighted as potential biomarkers for diagnosing endometritis. These findings indicate significant metabolic differences in blood metabolites between mares with endometritis and healthy mares, demonstrating the potential for diagnosis through metabolite analysis.

**Abstract:**

The aim of this study was to explore alterations in plasma metabolites among mares afflicted with endometritis. Mares were divided into two groups, namely, the equine endometritis group (*n* = 8) and the healthy control group (*n* = 8), which included four pregnant and four non-pregnant mares, using a combination of clinical assessment and laboratory confirmation. Plasma samples from both groups of mares were analyzed through untargeted liquid chromatography-tandem mass spectrometry (LC-MS/MS) metabolomics. A total of 28 differentially abundant metabolites were identified by screening and identifying differentially abundant metabolites and analyzing the pathway enrichment of differentially. Ten metabolites were identified as potential biomarkers for the diagnosis of endometritis in mares. Among them, seven exhibited a decrease in the endometritis groups, including hexadecanedioic acid, oleoyl ethanolamide (OEA), [fahydroxy(18:0)]12_13-dihydroxy-9z-octa (12,13-diHOME), deoxycholic acid 3-glucuronide (DCA-3G), 2-oxindole, and (+/-)9-HPODE, and 13(S)-HOTRE. On the other hand, three metabolites, adenosine 5′-monophosphate (AMP), 5-hydroxy-dl-tryptophan (5-HTP), and l-formylkynurenine, demonstrated an increase. These substances primarily participate in the metabolism of tryptophan and linolenic acid, as well as fat and energy. In conclusion, metabolomics revealed differentially abundant metabolite changes in patients with mare endometritis. These specific metabolites can be used as potential biomarkers for the non-invasive diagnosis of mare endometritis.

## 1. Introduction

Normally, in a healthy mare, the response to sperm resulting from mating can resolve spontaneously within 48 h [1]. However, inflammation caused by sperm of bacterial infection persisting beyond this timeframe can lead to endometritis. Endometritis significantly impacts mare fertility, with endometritis-induced pregnancy failures accounting for approximately 25–60% of total pregnancy losses [2].

The classification of endometritis varies. Based on its clinical presentation, it can be categorized into clinical and subclinical endometritis [3]. The clinical manifestations can be categorized as mucous, mucopurulent, or purulent endometritis [4]. The causes of inflammation can be divided into post–mating-induced endometritis (PMIE) and infectious endometritis [5]. Depending on the progression speed, it can be classified as acute or chronic endometritis [6,7]. Additionally, it is important to note that these categories are not absolute, as affected horses may transition between different categories or belong to multiple categories simultaneously. An accurate diagnosis of endometritis by veterinarians necessitates a comprehensive clinical assessment, coupled with various supplementary laboratory indicators, such as bacteriologic testing, neutrophil counts, and endometrial biopsy [7]. Nevertheless, collecting these samples for laboratory diagnosis typically involves invasive transvaginal procedures using swabs, cytobrushes, or biopsy-sample collectors [8]. These sampling techniques not only burden the operator but are also costly and carry the potential for additional infection or injury to the mare if not performed meticulously. Consequently, there is a pressing need to identify biological samples that are easily obtainable and entail minimal risk of secondary injuries to aid in the diagnosis of mare endometritis.

Metabolomics is centered on examining metabolites within biological systems [9]. Metabolites serve as the final outcomes of cellular regulatory mechanisms, and their expression levels often reflect the organism’s ultimate response to various alterations [10]. Blood comprises a substantial reservoir of metabolite data [11], and analyzing blood metabolites to identify diagnostic or prognostic biomarkers is emerging as a novel avenue of investigation [12]. Studies have now begun to focus on metabolite differences in the uterine secretions of mares with endometritis; coumaric acid, benzoic acid, and estragole are downregulated, and metabolites such as L-phenylalanine, glutamine, succinic acid, linoleate, and arachidonic acid are upregulated in samples of clinical endometritis compared to healthy uterine secretions [13]. Several studies have also identified metabolites such as non-esterified fatty acid, beta-hydroxybutyrate, and haptoglobin in the blood of cows with endometritis, which have the ability to predict and diagnose endometritis [14,15,16]. However, these studies were conducted in cattle, thus strengthening the rationale for our study in horses, which, to our knowledge, has not been attempted before. Additionally, the knowledge of innate immune mechanisms in mares is mostly limited to local, endometrial responses. While similar approaches have been explored in cows, they have not been addressed in mares.

Hence, the present study analyzed blood metabolites from healthy mares and mares suffering from endometritis using HPLC-QTOF-MS. We aimed to explore the distinctions between healthy and endometritis-affected mares from a metabolomic standpoint to elucidate the molecular underpinnings of endometritis. Furthermore, identifying specific metabolites that are differentially abundant in these groups holds promise for their application as potential biomarkers to diagnose endometritis.

## 2. Materials and Methods

### 2.1. Animal Selection and Grouping

This study was conducted at a large intensive breeding facility located in Shandan city, Gansu, where the sampling was concentrated between April and October. These mares were kept in captivity at intensive farming corrals under natural light conditions. Initially, any horses that had been administered any form of medication in the last three months were excluded. A group of mares aged 3–10 years with a parity of 2–7 and a body weight of 350 ± 20 kg was selected for the study. The diagnosis of endometritis followed the following criteria, with mares meeting both criteria: (1) failure to conceive after a minimum of two breeding cycles of continuous mating with a known fertile stallion and (2) rectal ultrasonography revealing inflammatory changes or fluid accumulation in the endometrium. These patients were identified as potential endometritis mares. These potential endometritis mares subsequently underwent double-protection cytology following the methodology outlined by Buczkowska, J. et al. and associated criteria [8]. Patients with ambiguous cytological results underwent supplementary bacterial culture, with the identification of one or more causative organisms of endometritis cultured on blood agar plates using a low-volume rinsing solution within 72 h. Mares with positive cytological- (presence of two or more neutrophils/400× microscope power field) or bacterial-culture results were classified into the endometritis group. To ensure minimal variability in sample collection, blood samples were collected from all mares identified as potential endometritis cases at the time of identification, but only those confirmed with endometritis were retained for subsequent studies. Additionally, mares diagnosed with endometritis underwent extensive veterinary clinical examinations to eliminate the possibility of coexisting pathological conditions, encompassing gastrointestinal system disorders, urinary tract infections, or dermatological issues. The control group comprised healthy horses and they were excluded from other disorders under all the examinations. A total of 8 healthy mares were selected for the control group, including 4 pregnant mares and 4 non-pregnant mares. Among the non-pregnant mares, 2 were in the estrus phase and 2 were in the post-estrus phase. Eight mares with endometritis were selected, and most of them were in the estrus or metestrus stages of the estrous cycle.

### 2.2. Plasma Sample Collection and Processing

Blood samples (10 mL each) were obtained from two groups of mares via jugular venipuncture using EDTA-K2 tubes (ritd., Taizhou, China). They were temporarily stored on ice (4 °C). Upon arrival at the laboratory, 5 mL of each sample was immediately centrifuged (1500× *g* for 15 min, 4 °C). The resulting plasma was separated and promptly frozen in liquid nitrogen for 30 min before being stored at –80 °C until further laboratory processing.

### 2.3. Metabolite Extraction

Twenty microliters of plasma was collected and mixed thoroughly with 120 microliters of 50% methanol for metabolite extraction. The mixture was allowed to stand for 10 min at 25 °C. Next, the extract was stored at −20 °C to induce protein precipitation. Subsequently, centrifugation was performed at 4000× *g* for 20 min, and the resulting supernatant was transferred to a 96-well plate. Ten microliters from each sample were prepared for quality control (QC). The samples were arranged in a randomized order, with QC samples specifically positioned at the beginning, middle, and end of the sequence to evaluate technical reproducibility. Prior to loading for analysis, all the samples, including the QC samples, were stored at −80 °C.

### 2.4. LC-MS/MS Analysis

LC-MS/MS analysis was conducted using a Thermo Scientific UltiMate 3000 HPLC system (Thermo Fisher Scientific Inc., Waltham, MA, USA) coupled with a Q Exactive mass spectrometer (Thermo Fisher Scientific Inc., Waltham, MA, USA). The chromatographic column used was an ACQUITY UPLC BEH C18 column (100 mm × 2.1 mm, 1.8 µm, Waters, UK). The column temperature was maintained at 35 °C, and the flow rate was set at 0.4 mL/min. In positive-ion mode, the mobile phase consisted of water (0.1% formic acid) as solution A and acetonitrile (0.1% formic acid) as solution B. The gradient profile for the solvent was as follows: 0~0.5 min, 5% B; 0.5~7 min, 5%~100% B; 7~8 min, 100% B; 8~8.1 min, 100%~5% B; and 8.1~10 min, 5% B.

The Q Exactive mass spectrometer was operated in positive- and negative-ion modes with a voltage of 5000 volts in positive- and –4500 volts in negative-ion modes. The ion source temperature was maintained at 650 °C. The ion source had a shielding gas pressure of 30 PSI. The pressures of gas 1 (auxiliary gas) and gas 2 (sheath gas) were both set to 60 psi. Precursor spectra were acquired at a resolution of 70,000 (range: 70–1050 m/z) to achieve an AGC target of 3 × 10^6^ with a maximum injection time of 100 ms. Mass spectra were acquired in data-dependent mode using data-dependent acquisition (DDA). Fragmentation spectra were obtained at a resolution of 17,500 to achieve an AGC target of 1 × 10^5^ with a maximum injection time of 80 ms. Instrument accuracy corrections were performed every 20 samples during the acquisition process, and quality control scans were conducted at intervals of 10 samples to correct systematic errors across the entire batch of experiments.

### 2.5. Data Analysis

The raw data were transformed into readable mzXML format using the MS Convert software(Version 3.0) from Proteowizard. Subsequently, peak extraction was carried out using XCMS software(Version 4.2.2), and rigorous quality control measures were applied to ensure the accuracy of peak extraction.

Following peak extraction and quality control, the primary metabolite information was gathered and subjected to comparative analysis with the online KEGG and HMDB databases to identify the primary metabolites. Secondary metabolites were identified by comparing the secondary fragmentation information with the local in-house database. Identification was achieved by meticulously matching the exact molecular mass data (*m*/*z*) of the sample with the data available in the database, resulting in the annotation of the respective metabolites. Only metabolites with a mass difference of less than 10 ppm compared to the database values were annotated. Furthermore, any features detected in less than 50% of the QC samples or less than 80% of the biological samples were systematically removed to ensure data integrity and reliability. The remaining peaks with missing values were then subjected to interpolation using the nearest-neighbor algorithm, further enhancing the data quality. Principal component analysis (PCA) was employed on the preprocessed datasets for outlier detection and assessment of batch effects. A robust LOESS signal-correction method based on QC data was applied to mitigate signal intensity drift over time by fitting it against the injection order. In addition, the relative standard deviation of metabolic signatures was calculated for all QC samples, and any signatures exhibiting a deviation greater than 30% were eliminated to maintain data quality and consistency.

To identify differences in metabolite concentrations between the two phenotypes, Student’s *t* tests were conducted. The resulting *p* values were adjusted for multiple testing using the FDR method, specifically the Benjamini-Hochberg procedure. For a more comprehensive analysis and discrimination of different variables between groups, supervised partial least squares discriminant analysis (PLS-DA) was employed, facilitated by metaX software(Version 2.86). In this analysis, VIP values were computed, and a VIP cutoff of 1.0 was applied to select the most significant and relevant features for further consideration. To determine the impact of pregnancy on the target differential metabolites, independent t−tests were conducted between pregnant and non-pregnant individuals in the healthy group using SPSS software (Version 27.0).

## 3. Results

### 3.1. Metabolomic Analysis of Mare Plasma

The total ion chromatograms (TICs) obtained from the analysis of the seven QC samples exhibited well-defined peak shapes and consistent peak distributions in both the positive- and negative-ion acquisition modes. This observation provides strong evidence of the stability and reliability of the assay (Figure S1). In addition, in the score plot generated via principal component analysis (PCA), the QC samples exhibited tight clustering, which highlighted the stability of the instrument, thus further demonstrating the credibility of the results (Figure S2).

**Figure 1:**
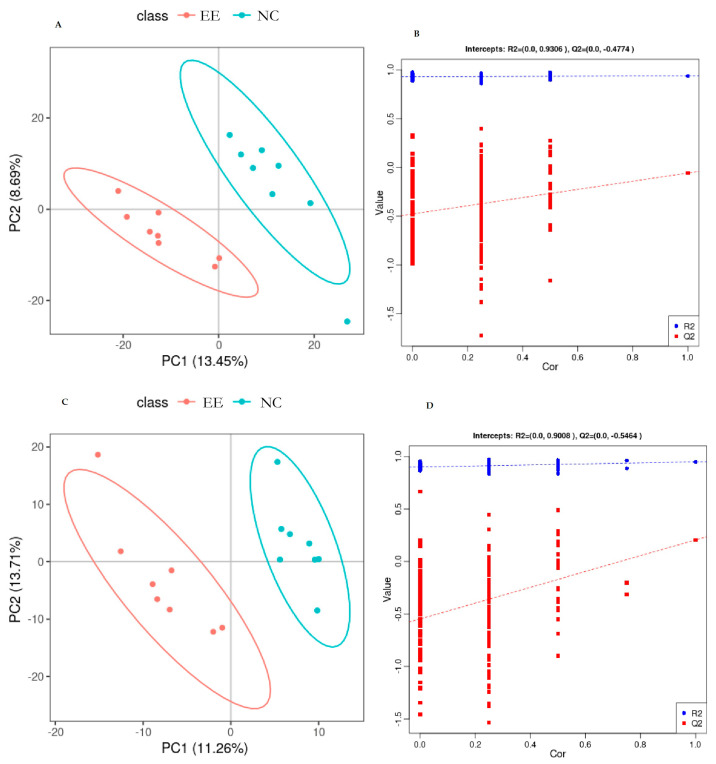
(**A**,**C**) Orthogonal partial least squares discriminant analysis (PLS−DA) score plot of mare plasma in positive- and negative-ion modes. (**B**,**D**) Permutation plot of PLS−DA in positive- and negative-ion modes. The blue dashed line represents R2, and the red dashed line represents Q2. EE represents the equine endometritis group, while NC represents the healthy control group.

In this study, the PLS-DA model was used to determine the overall differences in metabolic profiles between the two groups, which in turn led to the identification of differentially abundant metabolites in plasma between the control and endometritis groups. The results showed that under positive- and negative-model conditions, both the control and endometritis groups of samples were clearly separated according to the PLS-DA score plots, and no overlap was observed (Figure 1A,C). In Figure 1B,D, the results of the replacement tests in both the positive- and negative-ion modes are presented. The permutation test was iterated 200 times to assess statistical significance. Notably, the Q2 values, as determined by the stochastic model, are consistently lower than the Q2 values obtained from the original model during the replacement test. Furthermore, the intercept of the Q2 regression line intersects the vertical axis at a value less than zero. Additionally, the Q2 value of the stochastic model gradually decreased as the proportion of Y-variables replaced increased and as the retention of substitutions gradually decreased. These findings indicate that the model was not overfit and that the differentially abundant metabolite analysis was accurate.

### 3.2. Identification and Comparison of Differentially Abundant Metabolites

**Figure 2:**
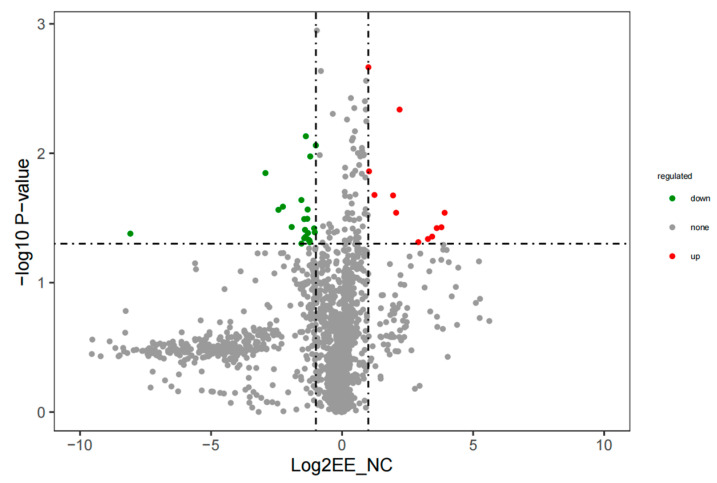
Volcano map of differentially abundant metabolites in the plasma of mares with endometritis and healthy mares. The horizontal axis represents the fold change, with dashed lines at 1 and −1. The vertical axis represents -log10(*p* value), with a dashed line at 1.3. Green dots represent significantly downregulated differential metabolites, red dots represent significantly upregulated differential metabolites, and gray dots represent differential metabolites with no significant changes.

**Figure 3:**
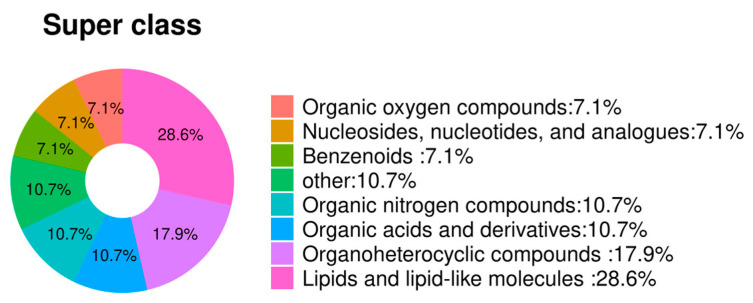
Classification of differentially abundant metabolites in the plasma of mares with endometritis and in that of healthy ones. The different color blocks in the graph indicate different taxonomic categories.

Based on the LC-MS/MS results, Table S1 lists the 25 distinct metabolites utilized for distinguishing between the endometritis group and control group (VIP > 1.0, *p* < 0.05), where metabolites with a fold change (endometritis group/control group) greater than 2 indicate upregulation and metabolites with a fold change (endometritis group/control group) less than 0.5 indicate downregulation. As shown in Figure 2, the raw *p* values calculated based on the differentially abundant metabolites are depicted as –log10 (*p* value). Notably, metabolites with y-coordinates exceeding 1.30, corresponding to –log10 (0.05), were considered to be significantly different. This threshold is used to identify metabolites that exhibit substantial variation between the two groups. The red points in the graph represent significant upregulation of metabolites; the green points indicate significant downregulation. The gray points indicate that the *p* value was less than 1.3, indicating that the difference was not significant. The differentially abundant metabolite types were analyzed and represented by a pie chart (Figure 3). Lipid and lipid-like molecules accounted for the greatest percentage (8, 28.6%), followed by organoheterocyclic compounds (5, 17.9%), organic acids and their derivatives (3, 10.7%), and organic nitrogen compounds (3, 10.7%).

**Figure 4:**
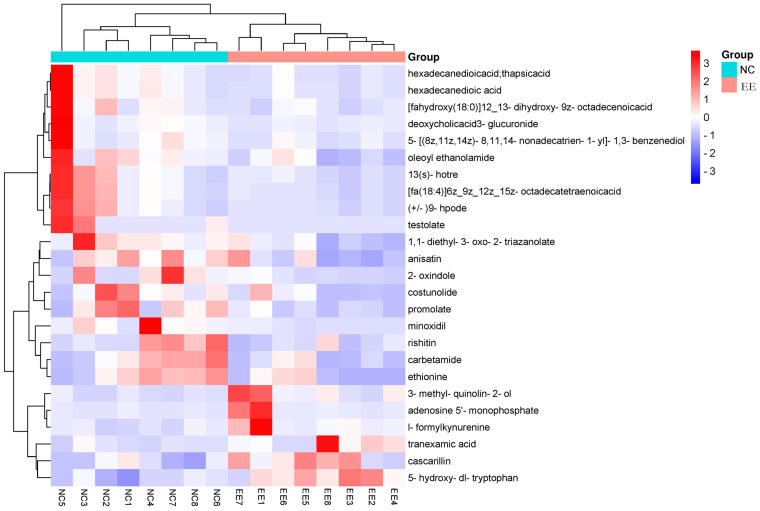
Heatmap of differentially abundant metabolites in the plasma of mares with endometritis and healthy mares. EE represents the equine endometritis group, while NC represents the healthy control group. The horizontal coordinates in the figure represent different mares, the vertical coordinates represent the differentially abundant metabolites, and the colors represent the relative levels of metabolites, where red indicates high content and blue indicates low content.

To obtain a more intuitive and clearer understanding of the variations in the 25 differentially abundant metabolites in the plasma of mares, the peak areas were analyzed by clustering. The results are presented in a heatmap (Figure 4). The blue group represents the control group, and the red group represents the endometritis group. The change in color represents the relative amount of expression.

**Figure 5:**
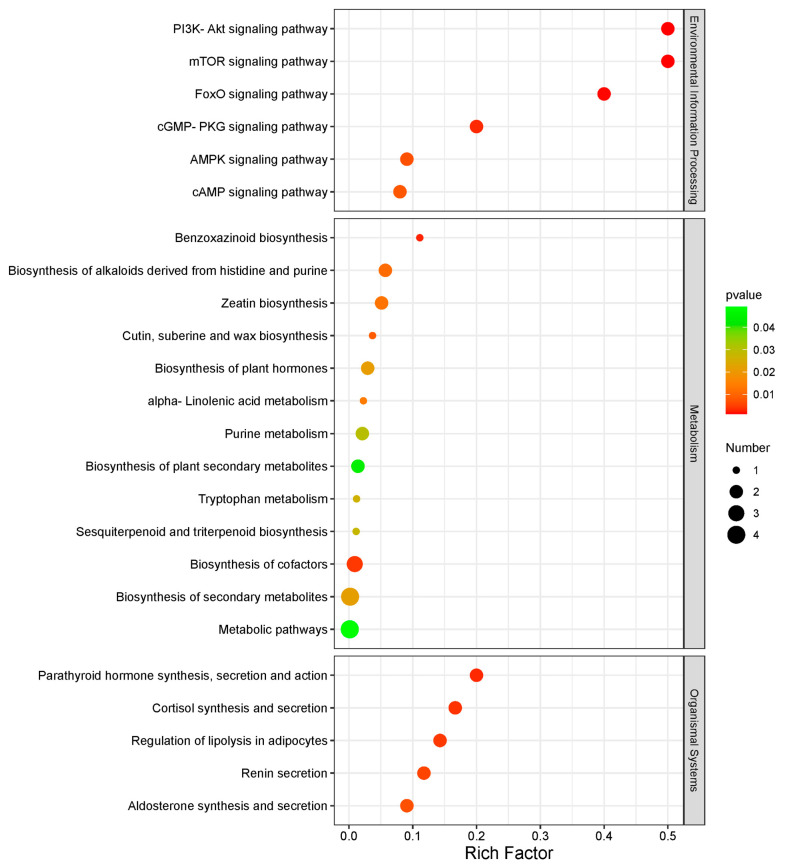
Bubble plot of KEGG enrichment pathways for differential metabolites in the plasma of mares with endometritis and in that of healthy ones. The size of the dots represents the number of enriched differentially abundant metabolites; the Rich Factor indicates the ratio of differentially abundant metabolites present in the KEGG pathway to the total number of metabolites in that specific KEGG pathway.

**Figure 6:**
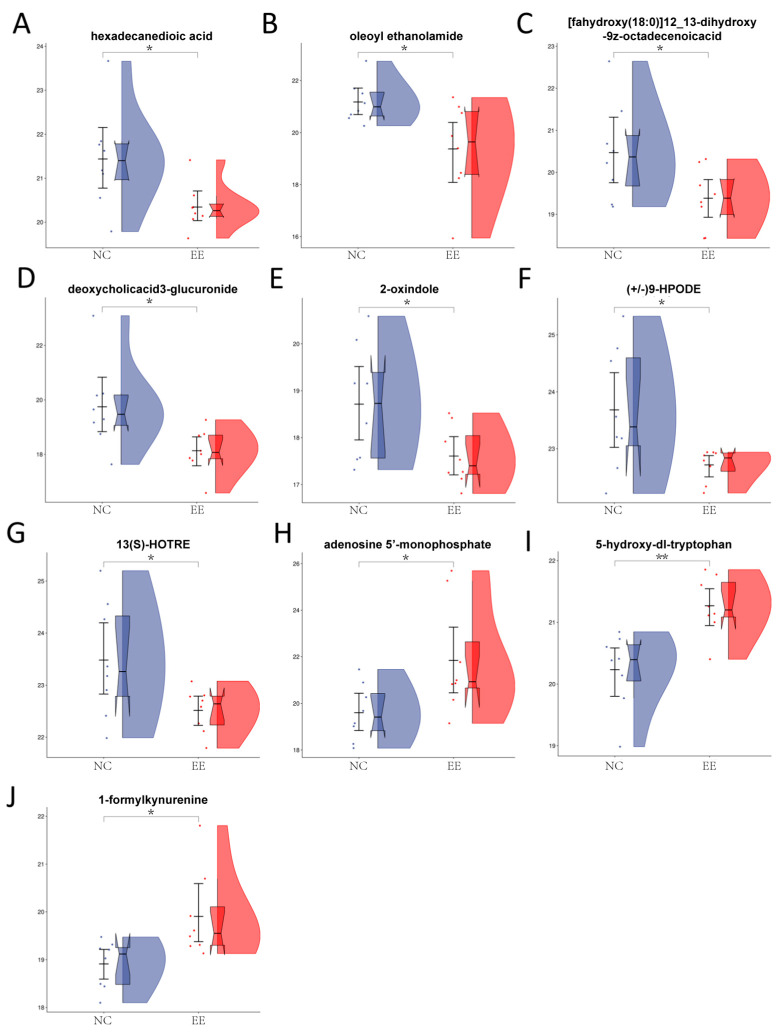
Relative peak areas of plasma-marker metabolites in mares with endometritis and healthy mares. (**A**) Hexadecanedioic acid. (**B**) Oleoyl ethanolamide, OEA. (**C**) [fahydroxy(18:0)]12_13-dihydroxy-9z-octa, 12,13-diHOME. (**D**) Deoxycholic acid 3-glucuronide (DCA-3G). (**E**) 2-oxindole. (**F**) (+/−)9-HPODE. (**G**) 13(S)-HOTRE. (**H**) adenosine 5′-monophosphate, AMP. (**I**) 5-hydroxy-dl-tryptophan, 5-HTP. (**J**) l-formylkynurenine. EE represents the equine endometritis group, while NC represents the healthy control group. “*” and “**” indicate statistical significance of the endometritis groups compared to the control group (“*” for *p* < 0.05, “**” for *p* < 0.01).

To further investigate the biomarkers, we searched the KEGG IDs (Kyoto Encyclopedia of Genes and Genomes Identifications) for a total of 24 differentially abundant metabolites. Differentially abundant metabolites were subjected to KEGG enrichment analysis, and the results were visualized using ggplot2 to illustrate the pathway enrichment outcomes at a significance level of *p* < 0.05, as shown in Figure 5. Notably, smaller *p* values indicate a greater degree of KEGG enrichment. Upon further analysis of the potential biological significance of these metabolites within these pathways and after excluding the impact of pregnancy in the control group (Table S2), it was determined that 10 of these metabolites hold promise as potential biomarkers for endometritis (Figure 6). Among these 10 metabolites, 7 exhibited a decreasing trend in the endometritis group, including hexadecanedioic acid, oleoyl ethanolamide, [fahydroxy(18:0)]12_13-dihydroxy-9z-octa, deoxycholic acid 3-glucuronide, 2-oxindole, (+/−)9-HPODE, and 13(S)-HOTRE. On the other hand, three metabolites, adenosine 5′-monophosphate, 5-hydroxy-dl-tryptophan, and l-formylkynurenine, exhibited increasing trends.

## 4. Discussion

Endometritis substantially impacts normal reproductive processes in mares, resulting in significant economic losses. Consequently, the need for precise and rapid diagnosis becomes imperative. While blood sampling is an invasive procedure, it is less invasive compared to other methods such as endometrial swabbing, and it can still provide valuable diagnostic information. The advantage of metabolomics lies in its ability to provide a comprehensive profile of metabolites. Previous studies have employed metabolomic techniques to identify plasma markers of endometritis in other animals [17]. In this study, plasma metabolite profiles from healthy mares were compared to those from mares with endometritis utilizing HPLC-QTOF-MS. Compared with other metabolomic techniques, HPLC-QTOF-MS offers superior sensitivity, resolution, and coverage of compounds [18]. It is also worth noting that the mares investigated in this study were all of reproductive age, meaning that healthy mares in the control group may develop endometritis as they age.

In our study results, we found that a majority of the pathways within the environmental information processing category appear to be closely associated with or involved in inflammatory and immune responses. The metabolism and organismal systems category also includes multiple pathways, such as tryptophan metabolism [19,20], alpha-linolenic acid metabolism [21,22], adipocyte regulation [23,24], purine metabolism [25,26], the metabolic pathway [27,28], cortisol synthesis and secretion [29,30], and renin secretion [31,32]; alternatively, these products may be related to the inflammatory response directly or indirectly.

It is widely recognized that tryptophan metabolism plays a pivotal role in developing and regulating inflammation. Inflammatory processes can lead to the metabolism of the essential amino acid L-tryptophan (Trp) through three distinct pathways: the kyn pathway, the 5-hydroxytryptamine (5-HT) pathway, and the indole-3-pyruvate (I3P) pathway [18]. Kyn is the most dominant metabolic pathway and accounts for approximately 95% of Trp degradation [33]. Kyn pathway activation is closely associated with immunosuppression, particularly in tumors or chronic inflammatory processes [34,35]. Specifically, the activation of the Kyn pathway results in the production of metabolites such as tryptophan, kynurenic acid, 3-hydroxykynurenine, and quinolinic acid. These factors can inhibit T-cell proliferation, induce apoptosis in certain immune cells, and promote the development and function of regulatory T cells (Tregs). L-Formylkynurenine serves as an intermediate metabolite within the Kyn pathway. In the presence of bacterial infection, LPS can stimulate the upregulation of IDO through signaling mediated by the NF-κB or p38-MAPK pathways [36]. This leads to the breakdown of L-tryptophan (L-TRP) into L-formylkynurenine by IDO. Subsequently, L-formylkynurenine undergoes further degradation into L-kynurenine (L-KYN), marking the first rate-limiting step in the kynurenine (KYN) pathway [37]. Upregulation of this pathway can result in local depletion of TRP, thereby inhibiting the immune response. However, certain bacteria can exploit KYN metabolism to evade the innate immune response. For instance, Pseudomonas aeruginosa, a common causative agent of mare bacterial endometritis [38], can utilize the KYN metabolism pathway to catabolize tryptophan, producing kynurenine. Kynurenine can scavenge reactive oxygen species (ROS) produced by neutrophils, thereby inhibiting the bactericidal activity of these immune cells. This process helps Pseudomonas aeruginosa evade the innate immune response, enhancing its survival during infections [39]. Thus, the significant increase in L-formylkynurenine among the differentially abundant metabolites in the endometritis groups may provide a new explanation for the persistence of inflammation. While this increase may represent an adaptive response of the host, it can also be exploited by pathogens in certain situations, leading to persistent infection.

Moreover, the level of the differentially abundant metabolite 5-hydroxytryptophan (5-HTP) was significantly elevated in the blood of endometritis patients. 5-HTP, which is a naturally occurring amino acid metabolite and one of the products derived from tryptophan, serves as a precursor for serotonin and directly influences serotonin production. Previous research has indicated that serotonin can effectively suppress inflammatory responses by regulating the activity of immune cells [40]. In the presence of bacterial infection, serotonin plays a role in inhibiting the production of TNF-α stimulated by LPS and increasing the release of IL-1β by peripheral blood mononuclear cells [41]. Furthermore, serotonin can also actively participate in the inflammatory response by affecting smooth muscle cells and endothelial cells. Specifically, in the context of inflammation, vascular smooth muscle cells may contract due to the binding of serotonin molecules to α-actinin [42]. This process can potentially impact the blood supply to inflamed areas and the functioning of inflammatory cells. Additionally, serotonin has the capacity to modulate leukocyte aggregation within inflamed blood vessels through its regulation of endothelial cells [43]. This modulation can lead to alterations in inflammation, vascular permeability, and immune cell activity. Therefore, the elevation of 5-HTP in peripheral blood may indicate an active response by the body to counteract uterine damage caused by factors such as local bacterial infection.

In our study, a noteworthy reduction in the levels of 2-oxindole was observed in the blood of the mares in the endometritis groups. Tryptophan undergoes degradation to indole through the action of tryptase [44], and the presence of 2-oxindole may indeed be relevant to the metabolism and synthesis of tryptophan. Previous studies have identified indole and its derivatives in various mammalian tissues, body fluids, and various natural products produced by plants, bacteria, and invertebrates [45]. Indole is a signaling molecule mediating communication across cells and even species [46]. In bacterium-induced inflammatory infections, indole plays diverse roles as a signaling molecule in both indole-producing and non-indole-producing bacteria. Specific examples related to common causative agents of mare commensal endometritis include the following: in Staphylococcus aureus, indole has been found to inhibit biofilm formation [47,48]. In Pseudomonas aeruginosa, indole can activate its active exocytosis function, enhance antibiotic tolerance, inhibit biofilm formation [49,50], or suppress the production [51] of virulence factors [50,52,53,54,55,56]. In Escherichia coli, indole is known to regulate antibiotic resistance [57].

In summary, elevated levels of indole can combat bacterial infections through various mechanisms: it inhibits the formation of biofilms, thereby compromising the bacteria’s defense layer and increasing susceptibility to antibiotic eradication or recognition and clearance by the host immune system. Additionally, indole modulates bacterial tolerance to antibiotics and their secretion systems, affecting their growth rate and pathogenicity. Importantly, indole can suppress the production of virulence factors, directly reducing the pathogenic potential of bacteria. Conversely, a decrease in indole levels may impair these defense mechanisms. Therefore, we speculate that the observed reduction in 2-hydroxyindole in our study could diminish the host’s ability to clear bacteria, potentially contributing to the persistence of pathogen-induced endometritis in mares.

A significant reduction in two differentially abundant metabolites related to linolenic acid metabolism was also found in this study. Linolenic acid and its metabolites may contribute to inflammation and immune responses. They can exert anti-inflammatory effects and diminish inflammatory responses by influencing cell membrane composition and function, controlling the release of inflammatory mediators, and participating in cell signaling pathways [58,59]. In this study, the levels of both the differentially abundant metabolites 9-HPODE and 13(S)-HOTRE were significantly lower in the endometritis groups.

9-HPODE is an oxidation product generated through a linoleic acid epoxygenase-dependent pathway [60]. It serves as an endogenous activator and ligand of the peroxisome proliferator-activated receptor protein (PPAR) [61]. Additionally, 9-HPODE stimulates interleukin-1β release from macrophages [62] and promotes monocyte/macrophage differentiation and the uptake of oxidized low-density lipoprotein (oxLDL) [63]. 13(S)-HOTRE belongs to the HODE (hydroxy-octadecenoic acid) family and is an active lipid metabolite. Alpha-linolenic acid (ALA) produces 13-HOTRE through the action of 12/15-LOX [64]. 13(S)-HOTRE exhibits a broad spectrum of biological activities [65]. During the inflammatory process of bacterial infection, 13(S)-HOTRE can activate the PPAR-γ receptor to deactivate the NLRP3 inflammatory vesicle complex, resulting in the downregulation of LPS-induced proinflammatory markers. Furthermore, it can exert anti-inflammatory effects by inhibiting autophagy and inducing apoptosis in LPS-challenged macrophages [66]. Therefore, we propose that the reduction in 9-HPODE and 13(S)-HOTRE in the endometritis groups may also be linked to the challenges of bacterial clearance and the persistence of inflammation in mare endometritis.

The persistent inflammatory process inevitably alters the body’s energy metabolism. This study also observed significant changes in five metabolites related to energy metabolism in the endometritis mares. Specifically, OEA, 12,13-diHOME, hexadecanedioic acid, and DCA-3G exhibited significant decreases in expression, while AMP significantly increased expression.

OEA is also an endogenous PPAR-α agonist. By activating PPAR-α, OEA inhibits the release of lipopolysaccharide (LPS)-induced proinflammatory cytokines, including TNF-α, IL-1β, COX-2, and IL-6, and mitigates the inflammatory response. OEA also hinders Toll-like receptor 4 (TLR4)-mediated responses by promoting PPAR-α signaling and suppressing the NF-κB signaling pathway, thereby reducing NF-κB-mediated inflammation. Simultaneously, OEA can regulate cellular signaling pathways, such as attenuating extracellular signal-regulated protein kinases 1 and 2 (ERK1/2) and reversing the STAT3 signaling cascade, thus inhibiting the activation of inflammatory responses [67,68].

12,13-diHOME is a phosphatidic acid generated through the hydrolysis of linoleic acid in response to cytochrome P450 (CYP) [69,70]. Regarding its role in immune and inflammatory regulation, 12,13-diHOME exhibits bidirectional effects. Its ability to trigger neutrophil chemotaxis at low concentrations [71,72] and inhibit the respiratory burst of these cells at high concentrations may lead to a limited inflammatory response [73]. 12,13-diHOME has also been demonstrated to enhance allergic responses [74] by inducing an allergic inflammatory response in a mouse model. This leads to increased infiltration of inflammatory cells and elevated expression of proinflammatory factors. These biological effects are likely mediated primarily through activating receptors such as PPARγ but may also involve other receptors such as TRPV1.

The relationship between AMP and AMPK is closely related, and a decrease in the intracellular ATP (adenosine triphosphate) concentration and an increase in the AMP concentration can lead to an increase in AMPK activity [75]. The activation of AMPK by AMP initiates a cascade of signaling pathways aimed at assisting the cell in restoring energy balance [76]. Therefore, in the context of chronic infections, cells may experience continuous stress, and elevated levels of AMP could serve as a mechanism for the body to manage prolonged energy depletion.

## 5. Conclusions

Taken together, the current findings indicate that for mares there may be a strong association with activation of the tryptophan metabolic pathway, inhibition of the linolenic acid metabolic pathway, and alterations in fat and energy metabolism and endometritis progression. Among the differentially abundant metabolites, AMP, 5-HTP, and l-formylkynurenine exhibited a significant increasing trend, and hexadecanedioic acid, OEA, 12,13-diHOME, DCA-3G, 2-oxindole,9-HPODE, and 13(S)-HOTRE exhibited a significant decreasing trend. Additionally, these data suggest that metabolomic analysis of persistent mare endometritis to identify new potential biomarkers is effective.

## Data Availability

Untargeted metabolomic data used in this publication have been deposited in the EMBL-EBI MetaboLights database with the identifier MTBLS9367. The complete data set can be accessed at https://www.ebi.ac.uk/metabolights/MTBLS9367 (accessed on 15 January 2024).

## Supplementary Materials

The following supporting information can be downloaded at: https://www.mdpi.com/article/10.3390/ani14131933/s1, Figure S1: Total ion chromatograms of plasma samples from mares with endometritis and healthy mares; Figure S2: PCA of LC-MS of plasma samples and plasma QC samples from mares with endometritis and healthy mares; Table S1: Identification of significantly different metabolites in the plasma of mares with endometritis and healthy mares; Table S2: Independent Samples T-Test for Differential Metabolites Between Pregnant and Non-Pregnant Mares in the Control Group.
