# Supplementary material for: Untargeted Metabolomic Analysis Reveals Plasma Differences between Mares with Endometritis and Healthy Ones
Source: Animals (Basel). 2024 Jun 29;14(13):1933. doi: 10.3390/ani14131933 (PMC11240781; doi:10.3390/ani14131933)
Supplement: Supplementary file 1 [file animals-14-01933-s001.zip › animals-3014353-supplementary.pdf]

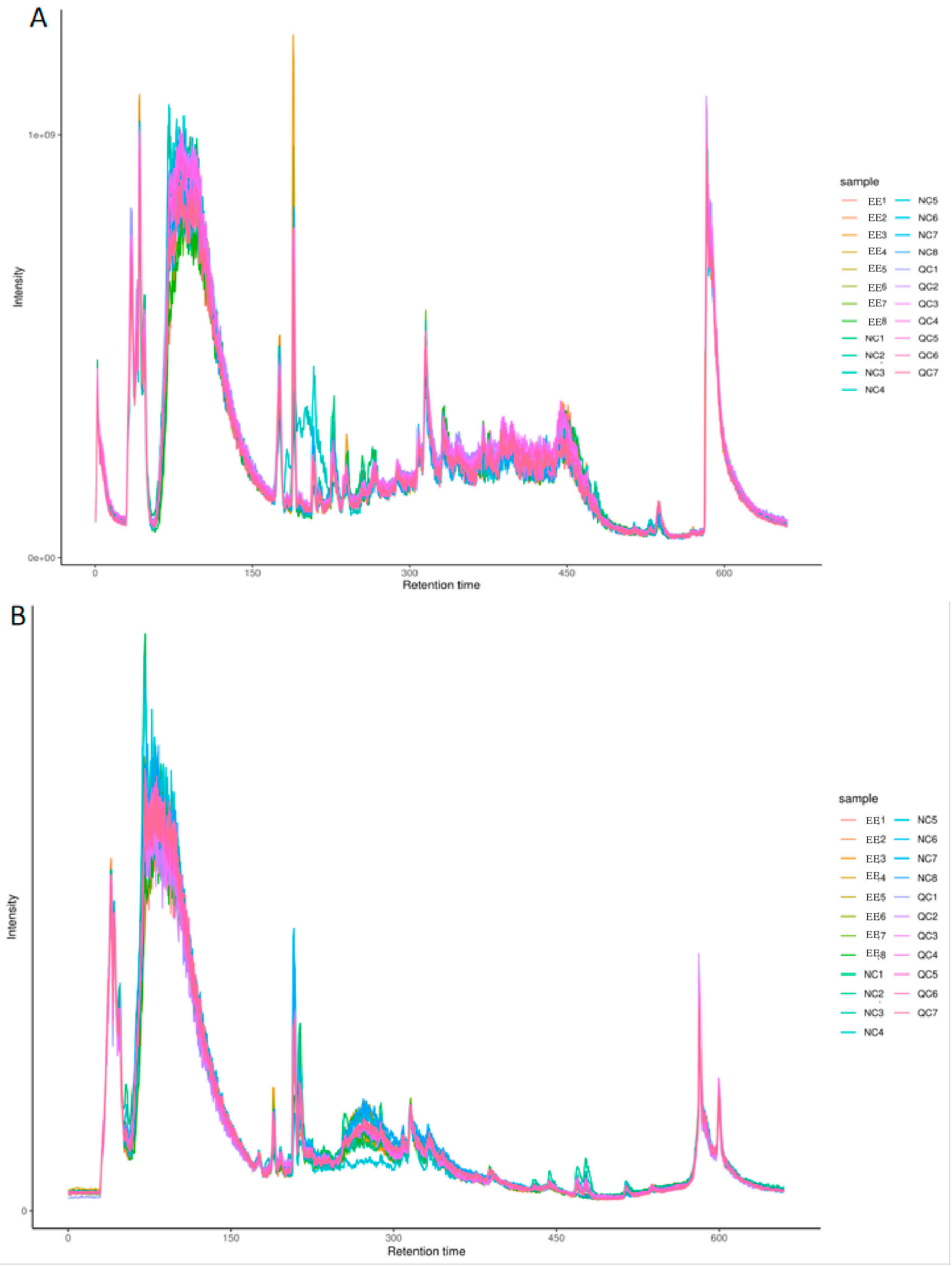

Figure S1. Total ion chromatograms of plasma samples from mares with endometritis and healthy mares. (a) Total ion chromatograms of samples in positive ion mode. (b) Total ion chromatograms of samples in negative ion mode.

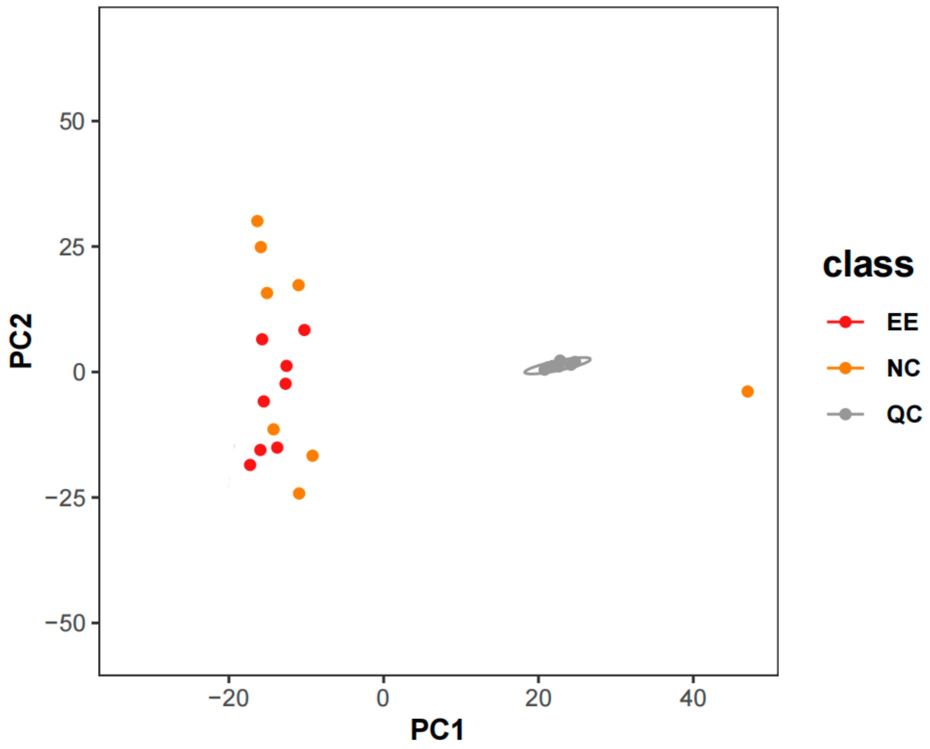

（a）

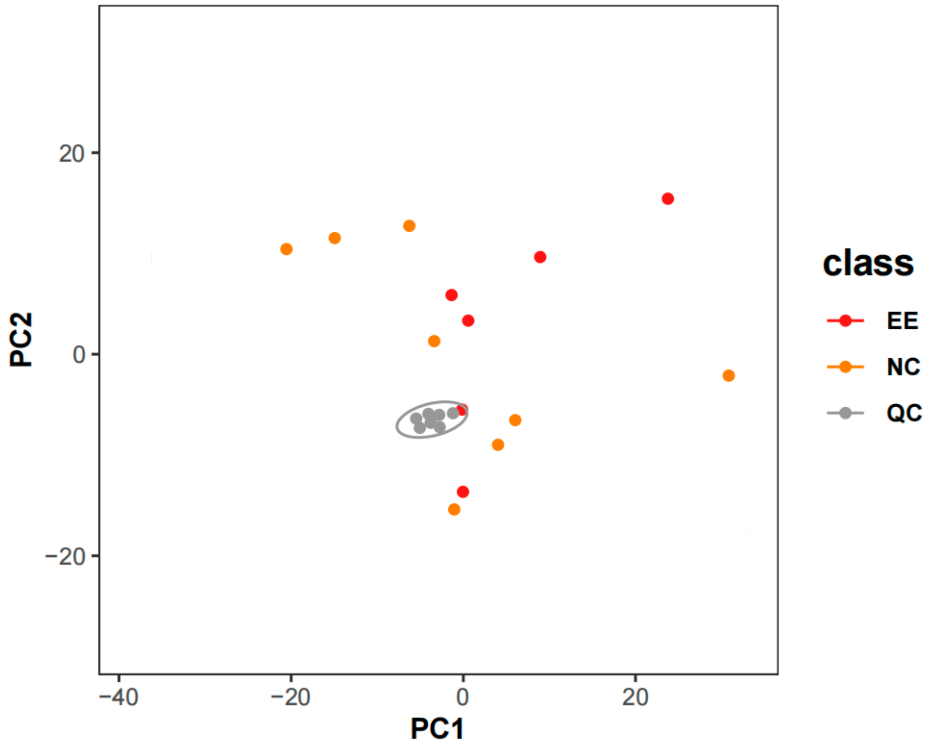

(b)

Figure S2. PCA of LC-MS of plasma samples and plasma QC samples from mares with endometritis and healthy mares. (a) PCA of QC samples in positive ion mode. (b) PCA of QC samples in negative ion mode. Orange and red circles represent plasma samples, and gray circles represent QC samples.

PCA, principal component analysis; QC, quality control.

Table S1. Identification of significantly different metabolites in the plasma of mares with endometritis and healthy mares.

ID	Metabolite name	M/Z	RT（min）	Fold change (EE/NC)	p.value	VIP	regulated
neg-4.084_320.19851	testolate	320.19851	4.084	0.003695007	0.041800085	1.612103312	down
pos-3.34_209.12749	minoxidil	209.12749	3.34	0.131640658	0.014216923	2.031346174	down
neg-3.994_568.32454	deoxycholicacid3-glucuronide	568.32454	3.994	0.185615357	0.027361945	1.898232909	down
pos-5.455_370.28627	5-[(8z,11z,14z)-8,11,14-nonadecatrien-1-yl]-1,3-benzenediol	370.28627	5.455	0.26359165	0.037130636	1.761805634	down
pos-5.074_286.21352	hexadecanedioicacid;thapsicacid	286.21352	5.074	0.340616281	0.022983038	1.990770224	down
pos-4.243_236.11547	carbetamide	236.11547	4.243	0.341492664	0.049899357	1.714348517	down
neg-5.075_286.21411	hexadecanedioic acid	286.21411	5.075	0.367215579	0.032244541	1.942484515	down
pos-3.685_133.05258	2-oxindole	133.05258	3.685	0.367284453	0.045348844	1.998180189	down
pos-5.586_232.14574	costunolide	232.14574	5.586	0.375200045	0.039056584	1.820041196	down
pos-5.719_314.24486	[fahydroxy(18:0)]12_13-dihydroxy-9z-octadecenoicacid	314.24486	5.719	0.380331318	0.044236293	1.789556106	down
pos-5.353_276.20807	[fa(18:4)]6z_9z_12z_15z-octadecatetraenoicacid	276.20807	5.353	0.398035785	0.03209323	1.963427303	down
pos-6.977_325.29723	oleoyl ethanolamide	325.29723	6.977	0.401987665	0.027209465	1.992307735	down
neg-5.351_312.22977	(+/-)9-hpode	312.22977	5.351	0.405206099	0.041340086	1.934923549	down
pos-5.352_294.21865	13(s)-hotre	294.21865	5.352	0.4117094	0.046551871	1.813807747	down
pos-3.449_293.16238	promolate	293.16238	3.449	0.421890578	0.047279616	2.023561631	down
pos-0.642_163.06616	ethionine	163.06616	0.642	0.426160926	0.04872543	1.702133966	down
pos-4.532_222.16127	rishitin	222.16127	4.532	0.477261841	0.038040406	2.015402851	down
neg-3.627_328.11551	anisatin	328.11551	3.627	0.485321993	0.040784344	1.9286722	down
neg-3.607_132.07753	1,1-diethyl-3-oxo-2-triazanolate	132.07753	3.607	0.498899373	0.008692739	2.265804541	down
pos-3.006_220.08435	5-hydroxy-dl-tryptophan	220.08435	3.006	2.005667236	0.002164826	2.543389443	up
neg-4.003_408.21457	cascarillin	408.21457	4.003	2.040920528	0.013793797	2.189434979	up
pos-2.983_236.07923	l-formylkynurenine	236.07923	2.983	2.353950418	0.020997497	2.031356902	up
pos-0.796_157.10991	tranexamic acid	157.10991	0.796	3.849551545	0.021177655	1.979712878	up
pos-1.169_157.10991	tranexamic acid	157.10991	1.169	4.174204574	0.028817271	1.880226263	up
pos-0.82_159.06792	3-methyl-quinolin-2-ol	159.06792	0.82	4.574242577	0.004595164	2.332896437	up
pos-2.05_159.06795	3-methyl-quinolin-2-ol	159.06795	2.05	7.510777988	0.048631612	1.714456215	up
pos-0.793_347.06213	adenosine 5’-monophosphate	347.06213	0.793	10.828058	0.044043505	1.662230622	up
pos-1.175_347.06219	adenosine 5’-monophosphate	347.06219	1.175	12.21853434	0.03786857	1.718559451	up

MZ, Mass-to-charge ratio of identified metabolic ions; RT, retention time; VIP, variable importance in the projection.

Table S2. Independent Samples T-Test for Differential Metabolites Between Pregnant and Non-Pregnant Mares in the Control Group

		Levene's Test for		T-test for Equality of Means						
		F	Levene's Test for	t	df	Sig. (2-tailed)	Mean Difference	Std. Error Difference	Confidence Interval of the Lower	Upper
hexadecanedioic acid	Assumed equal variances	5.136	0.064	-0.992	6	0.360	-2749407.01	2772057.72	-9532387.90	4033573.87
oleoyl ethanolamide	Not assumed equal variances			-0.992	3.172	0.391	-2749407.01	2772057.72	-11307279.89	5808465.87
8:0)]12_13-dihydroxy-9z-octa	Assumed equal variances	4.085	0.090	-0.217	6	0.835	-310205.24	1426522.91	-3800781.06	3180370.58
deoxycholicacid3-glucuronid	Not assumed equal variances			-0.217	3.476	0.840	-310205.24	1426522.91	-4517984.44	3897573.97
2-oxindole	Assumed equal variances	2.946	0.137	-0.642	6	0.545	-938312.71	1461811.74	-4515237.18	2638611.75
(+/-)9-hpode	Not assumed equal variances			-0.642	3.791	0.558	-938312.71	1461811.74	-5086716.01	3210090.59
13(s)-hotre	Assumed equal variances	7.096	0.037	-1.013	6	0.350	-2086735.29	2059828.87	-7126954.97	2953484.39
5-hydroxy-dl-tryptophan	Not assumed equal variances			-1.013	3.058	0.384	-2086735.29	2059828.87	-8571931.82	4398461.23
adenosine 5'-monophosphat	Assumed equal variances	0.619	0.461	0.232	6	0.824	89852.79	386933.74	-856939.96	1036645.55
l-formylkynurenine	Not assumed equal variances			0.232	5.079	0.825	89852.79	386933.74	-900147.09	1079852.68
hexadecanedioic acid	Assumed equal variances	3.776	0.100	-1.016	6	0.349	-9397540.04	9248074.60	-32026763.38	13231683.29
oleoyl ethanolamide	Not assumed equal variances			-1.016	4.327	0.363	-9397540.04	9248074.60	-34326826.22	15531746.13
8:0)]12_13-dihydroxy-9z-octa	Assumed equal variances	4.230	0.085	-1.053	6	0.333	-8723037.06	8281167.99	-28986325.15	11540251.02
deoxycholicacid3-glucuronid	Not assumed equal variances			-1.053	4.025	0.351	-8723037.06	8281167.99	-31659431.06	14213356.93
2-oxindole	Assumed equal variances	2.051	0.202	-1.223	6	0.267	-376869.17	308276.39	-1131194.33	377455.99
(+/-)9-hpode	Not assumed equal variances			-1.223	4.801	0.278	-376869.17	308276.39	-1179281.59	425543.25
13(s)-hotre	Assumed equal variances	1.718	0.238	0.462	6	0.661	111054.93	240547.10	-477542.63	699652.49
5-hydroxy-dl-tryptophan	Not assumed equal variances			0.462	4.673	0.665	111054.93	240547.10	-520555.10	742664.96
adenosine 5'-monophosphat	Assumed equal variances	4.276	0.084	-1.645	6	0.151	-169253.62	102867.33	-420960.90	82453.66
l-formylkynurenine	Not assumed equal variances			-1.645	3.361	0.189	-169253.62	102867.33	-477589.63	139082.40
